# Tumor-derived alpha-fetoprotein impairs the differentiation and T cell stimulatory activity of human dendritic cells

**DOI:** 10.1186/2051-1426-2-S3-P229

**Published:** 2014-11-06

**Authors:** Angela D Pardee, Jian Shi, Lisa H Butterfield

**Affiliations:** 1 Department of Medicine, University of Pittsburgh, Pittsburgh, PA, USA; 2 Departments of Medicine, Immunology and Surgery and University of Pittsburgh Cancer Institute, Pittsburgh, PA, USA

## 

Several tumor-derived factors have been implicated in DC dysfunction in cancer patients. Alpha-fetoprotein (AFP) is an oncofetal antigen that is highly expressed in abnormalities of prenatal development and several epithelial cancers, including hepatocellular carcinoma (HCC). In HCC patients exhibiting high levels of serum AFP, we have observed a lower ratio of myeloid-to-plasmacytoid circulating DC compared to patients with low serum AFP levels and healthy donors, suggesting that AFP alters DC differentiation in vivo. To test the effect of AFP on DC differentiation in vitro, peripheral blood monocytes from healthy donors were cultured in the presence of cord blood-derived normal AFP (nAFP) or HCC tumor-derived AFP (tAFP), and DC phenotype and function was assessed. Although the nAFP and tAFP isoforms only differ at one carbohydrate group, low (physiological) levels of tAFP, but not nAFP, significantly inhibited DC differentiation. tAFP-conditioned DC expressed diminished levels of DC maturation markers, retained a monocyte-like morphology, exhibited limited production of inflammatory mediators, and failed to induce robust T cell proliferative responses. Mechanistic studies revealed that the suppressive activity of tAFP is dependent on the presence of low molecular weight (LMW) species that *i) *co-purify with tAFP, and *ii) *are abundant in the LMW fractions of both tumor and non-tumor cell lysates. These data reveal the unique ability of tAFP to serve as a chaperone protein for ubiquitous LMW molecules, which function cooperatively to impair DC differentiation and function. Therefore, novel therapeutic approaches that antagonize the regulatory properties of tAFP will be critical to enhance immunity and improve clinical outcomes.

